# Complications and range of motion of patients with an elbow dislocation treated with a hinged external fixator: a retrospective cohort study

**DOI:** 10.1007/s00068-022-02013-x

**Published:** 2022-06-25

**Authors:** Bart Van Tunen, Esther M. M. Van Lieshout, Konrad Mader, Dennis Den Hartog

**Affiliations:** 1 Trauma Research Unit, Department of Surgery, Erasmus MC, University Medical Center Rotterdam, P.O. Box 2040, 3000 CA Rotterdam, The Netherlands; 2 Division Hand, Forearm and Elbow Surgery, Department of Trauma and Orthopaedic Surgery, University Medical Center Hamburg-Eppendorf, Hamburg, Germany

**Keywords:** Elbow dislocation, Persistent instability, Hinged external fixator, Fixator-related complications, Range of motion, Centralization

## Abstract

**Purpose:**

Elbow dislocations are at risk for persistent instability and stiffness of the joint. Treatment with a hinged external fixation provides elbow joint stability, and allows early mobilization to prevent stiffness. Mounting a hinged elbow fixator correctly, however, is technically challenging. The low incidence rate of elbow dislocations with persistent instability suggests that centralization would result in higher surgeon exposure and consequently in less complications. This study aimed to investigate the results of treatment of elbow dislocations with a hinged elbow fixator on the rate of complications, range of motion, level of pain and restrictions in activities of daily living.

**Methods:**

A retrospective observational cohort study in a level I trauma center, in which the majority of patients was treated by a dedicated elbow surgeon, was performed. All patients of 16 years or older treated with a hinged external elbow fixator between January 1, 2006 and December 31, 2017 were included. The fixator could be used (1) for the treatment of persistent instability in acute/residual simple and complex dislocations or (2) as revision surgery to treat joint incongruency or a stiff elbow. Patient and injury characteristics, details on treatment, complications, secondary interventions, and range of motion were extracted from the patients’ medical files.

**Results:**

The results of treatment of 34 patients were analyzed with a median follow-up of 13 months. The fixator was removed after a median period of 48 days. Fixator-related complications encountered were six pintract infections, one redisclocation, one joint incongruency, one muscle hernia, and one hardware failure. The median range of motion at the end of follow-up was 140° flexion, 15° constraint in extension, 90° pronation, and 80° supination.

**Conclusion:**

A hinged elbow fixator applied by a dedicated elbow surgeon in cases of elbow instability after elbow dislocations can result in excellent joint function. Fixator-related complications are mostly mild and only temporary.

## Introduction

The elbow joint is the second most commonly dislocated joint in adults with an incidence rate of 6.1 per 100,000 persons per year [1]. In some cases, the elbow is persistently unstable due to concomitant periarticular fractures and gross ligamentous and muscle damage. In case of surgical repair, the primary therapeutic goal after surgery is to preserve joint motion while protecting the healing ligaments. Early active mobilization results in greater range of motion, a faster recovery, and faster return to work [2]. Hinged external fixation is an important supplement to open reduction and internal fixation (ORIF) and ligament repair for selected unstable elbow injuries. Because the elbow joint closely approximates a simple hinge, external fixation with a hinged device will keep the elbow reduced while allowing controlled hinged and rotational motion which corresponds to the natural elbow joint. This protects the elbow against valgus and varus stress and allows flexion and extension, whereby the ligaments can heal without additional reconstruction [3].

Elbow dislocations can be classified as simple or complex. Simple dislocations are characterized by ligamentous damage and the absence of fractures, while complex dislocations are either associated with gross ligamentous and muscular damage or with fractures of the radial head, coronoid process, or olecranon [4]. Both types of elbow dislocation are at risk for persistent instability, which may be a prelude to chronic elbow instability. Also, dislocated joints are at risk for the development of a stiff elbow due to prolonged immobilization, resulting in a restricted range of motion [2, 5]. Treatment of chronic elbow instability and elbow stiffness is challenging.

Over the last decennium, treatment with a hinged external fixator has been a permanent consideration for elbow dislocations. Many other treatment options (ORIF with screws and plates, intramedullary nailing, free vascularized bone grafting, cross-pinning of the elbow, total elbow arthroplasty) have been examined in previous series. Outcomes were not satisfying however, while rates of complications like infections, triceps insufficiency, pin loosening, injury to adjacent neurovascular structures, cellulitis, and loss of reduction remain high [6–10].

Hinged external fixation for six till eight weeks could provide a faster recovery by providing joint stability and allowing for early mobilization. It plays an important role in the treatment of elbow dislocations by preventing stiffness of the joint. Over the last years, multiple studies showed a significant improve in the outcome of the range of motion by applying a hinged external fixator for elbow dislocation [3, 6, 7, 11–13]. However, mounting a hinged elbow fixator is a technically challenging procedure in which, especially, the center of rotation is hard to determine [14, 15]. Although other studies already mentioned that higher surgeon volume was associated with improved outcomes and decreased adverse events, the effect of an experienced surgical team on the outcome of complex elbow surgery has not been reported [16]. The low incidence rate of elbow dislocations with persistent instability suggests that centralization would result in higher surgeon exposure and consequently in less complications.

The aim of this study was to investigate the effects of treatment of elbow dislocations with persistent instability using a hinged elbow fixator on the rate of complications and secondary interventions, range of motion, level of pain, and restrictions in activities of daily living. The study was performed in a medical center in which the majority of patients was treated by a dedicated elbow surgeon with extensive experience in the application of hinged elbow fixators.

## Methods

The study was exempted by the local Medical Research Ethics Committee (number MEC-2018–1527). The STROBE Statement checklist for cohort studies was followed in the writing of this manuscript [17].

**Fig. 1:**
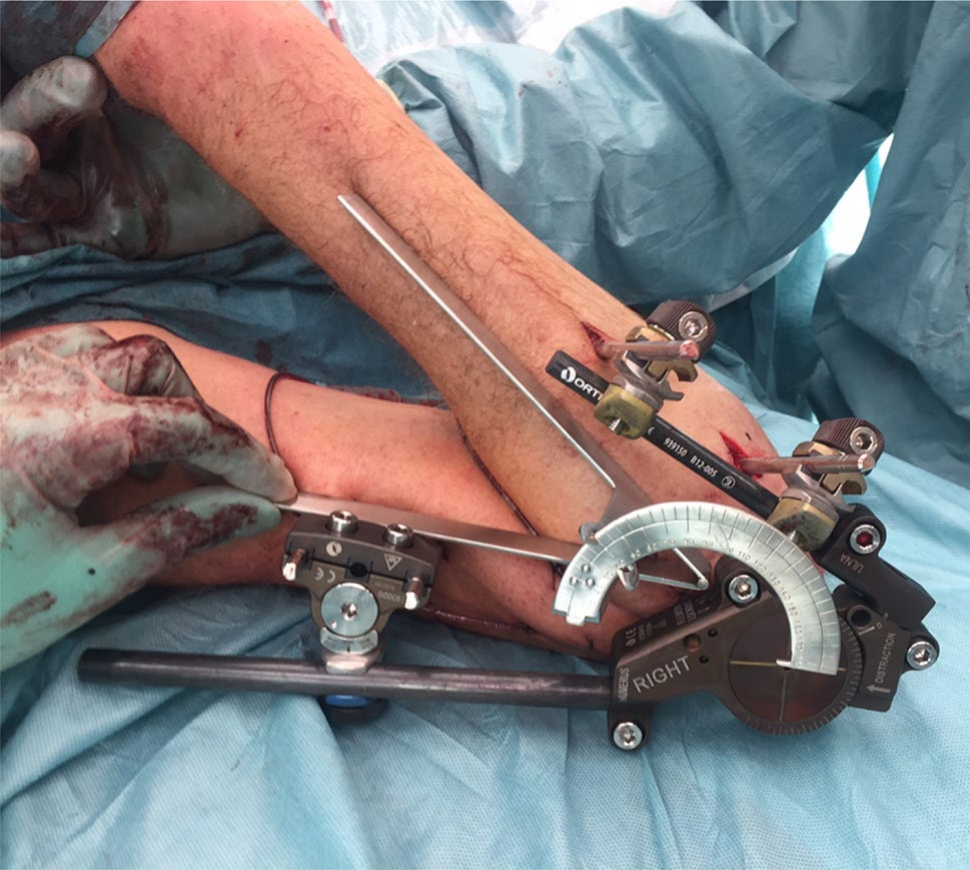
Orthofix® (Galaxy) elbow fixator

**Fig. 2:**
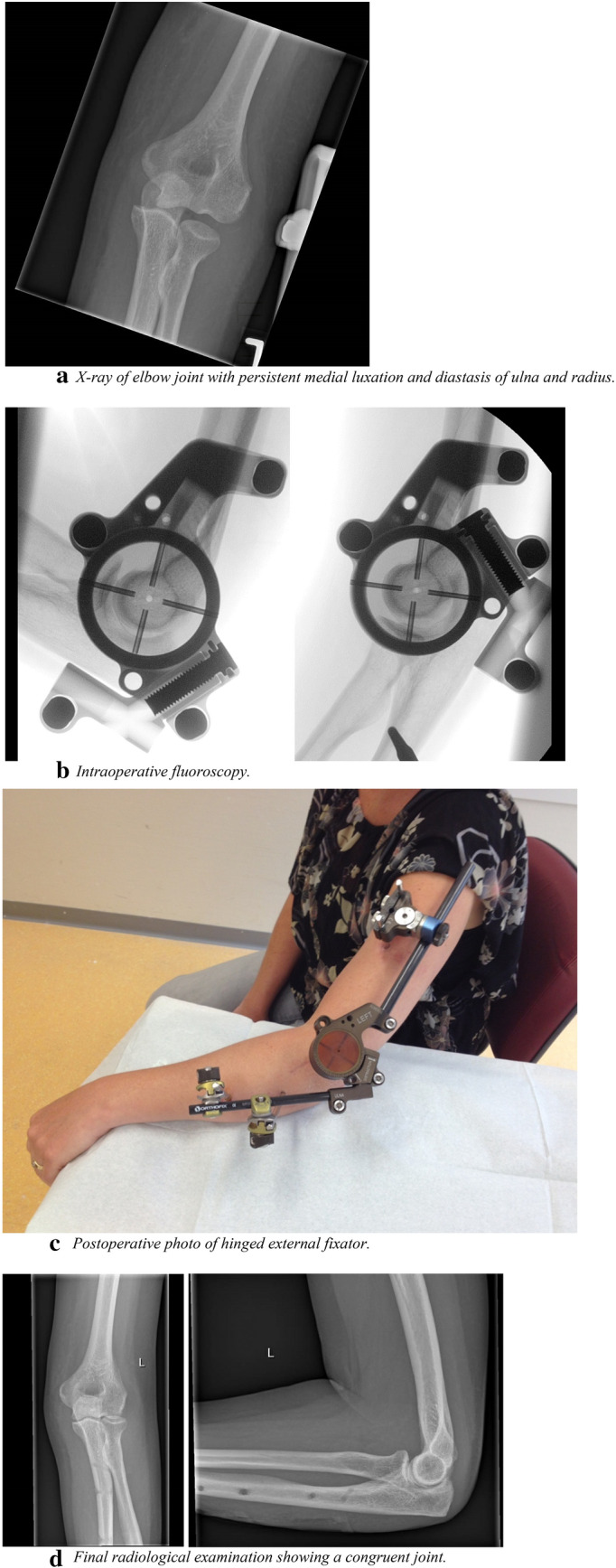
Radiological findings of a typical case. Hereby we present a case from a patient with a simple elbow dislocation with persistent luxation after external fixation elsewhere, after a low-energy trauma caused by a fall on March 8th, 2012. **a** X-ray of elbow joint with persistent luxation and diastasis of ulna and radius. We removed the static fixator, performed an extended arthrolysis and anatomic reduction of the elbow joint, followed by applying a dynamic hinged elbow fixator on May 15th, 2012 (68 days after trauma), resulting in congruent elbow joint in flexion and extension during surgery. **b** Intraoperative fluoroscopy images. **c** Postoperative photo of hinged external fixator. After 8 weeks, the dynamic fixator was removed. **d** The treatment resulted in a congruent anatomically joint seen on final radiological examination. At last follow-up clinical result was excellent with a flexion–extension arc of 140° and pronation-supination arc of 180°. The postoperative course was uneventful, and the patient was discharged from follow-up after 7.3 months

**Fig. 3:**
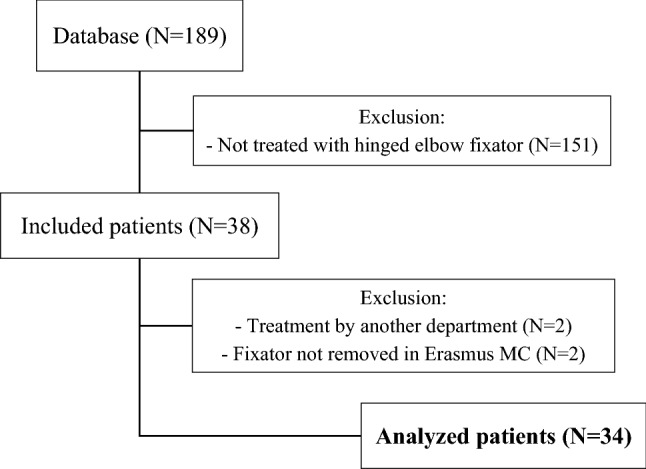
Study flow chart

A retrospective observational cohort study was conducted in a level I trauma center. Patients were identified from the medical files using the following codes of surgery: elbow arthrolysis, elbow arthrotomy, and treatment of recent elbow dislocation. Patients aged 16 years or older who were treated with a hinged elbow fixator (Orthofix**®** (Galaxy) elbow fixator; Orthofix International, Bussolegno, Italy), between January 1, 2006 and December 31, 2017 were included (Fig. 1). Three groups of patients with either simple or complex elbow dislocations were managed with a hinged external fixator: (1) acute elbow instability following trauma, managed within 2 weeks; (2) acute elbow instability following trauma, managed after more than 2 weeks (Fig. 2); (3) persisting instability after arthrolysis in patients with a stiff elbow. The hinged external elbow fixator had to be mounted as well as removed at the participating hospital. Patients who were treated by another department without consultation of a dedicated elbow trauma surgeon were excluded.

Elbow instability was defined by immediate redislocation after reduction, and/or (sub)luxation with fluoroscopy. With our strategy we aimed on natural healing of the ligaments (by keeping the joint in anatomic reduction, allowing motion in a stable joint due to the fixator). We restored radial head fractures with screw fixation after anatomic reduction and we fixated only type 2 and 3 coronoid fractures, especially with antero-medial extension with involvement of the insertion of the anterior bundle of the medial collateral ligament (AMCL). Avulsion fractures were not fixated; even type 1 coronoid was not fixated.

All patients visited the outpatient clinic on a regular basis during follow-up; normally in week 1, 3, 6, 12, 24 and 52 after surgery. The follow-up ended when the patient was discharged from outpatient visit. Primary outcomes were the rate, as well as the type and severity of complications. Secondary outcomes were the rate of secondary interventions, the range of motion, the level of pain, restrictions in activities of daily living at discharge from clinical follow-up as well as the experience of the surgical team. The team was classified as experienced when the senior or supervising surgeon was a trained elbow fixator surgeon who attended multiple training programs and courses specific for this type of surgery. Patient and injury characteristics as well as details on treatment were extracted from the patients’ medical files.

Data were analyzed using the Statistical Package for the Social Sciences (SPSS) version 24.0 (SPSS, Chicago, Ill., USA). Normality of continuous data was tested using the Shapiro Wilk test. Descriptive analysis was performed to describe baseline characteristics (intrinsic, injury-related, and intervention-related variables) and outcome measures. Continuous data are reported as median with percentiles (non-parametric data) and categorical data are reported as number with percentage.

## Results

A total of 189 patients underwent elbow surgery during the study period, of whom 38 had been treated with a hinged elbow fixator. Four patients were excluded; two patients were treated by another department without consultation of a dedicated elbow trauma surgeon and in two other patients the fixator was removed in another hospital. A total number of 34 patients with a median follow-up of 13 (P_25_-P_75_ = 8–21) months were analyzed (Fig. 3). 24 (71%) operations were performed by a surgical team that was classified as experienced and treated 8 patients (89%) with a simple elbow dislocation and 16 (64%) patients with a complex dislocation. The hinged external fixator surgery was performed a median of seven (P_25_-P_75_ = 0–20) days after trauma and was removed a median of 48 (P_25_-P_75_ = 42–57) days later.

An overview of patient and injury characteristics, with a subdivision into simple and complex dislocations, is shown in Table 1. The median age was 49 (P_25_-P_75_ = 29–61) years in a predominantly male cohort (*n* = 20, 59%). Twentyfive (74%) patients suffered a complex dislocation, of which 21 were managed acutely. In 19 (56%) patients, the mechanism of injury was valued as low energy.

**Table 1:** Patient and injury characteristics of patients with a simple or complex elbow dislocation that was treated with a hinged fixator

	Total group (*N* = 34)	Simple dislocation (*N* = 9)	Complex dislocation (*N* = 25)
Male gender	20 (59%)	4 (44%)	16 (64%)
Age (years)	49 (29–61)	53 (38–66)	47 (28–59)
Simple dislocation	9 (26%)	9 (100%)	0 (0%)
Right side injured	16 (47%)	3 (33%)	13 (52%)
Dominant side injured*	6 (43%)	1 (50%)	5 (42%)
Low-energy trauma	19 (56%)	7 (78%)	12 (48%)

Data are shown as *n* (%) or as median (P_25_–P_75_)

^*^Data were missing for 7 patients with a simple dislocation and 13 patients with a complex dislocation

Nine (26%) patients developed one or more fixator-related complications and eight (24%) patients required one or more secondary interventions due to these fixator-related problems (Table 2). Only one patient with a simple dislocation developed a complication and required a secondary intervention, being a joint incongruence for which the fixator was realigned. The other eight patients all had a complex dislocation. The complications in this group were pintract infections (*n* = 6 patients), redislocation (*n* = 1), muscle hernia (*n* = 1) and hardware failure (*n* = 1), for which treatment with antibiotics (*n* = 1), incision and drainage (*n* = 4), fixator realignment (*n* = 1) and fixator exchange (*n* = 2) was carried out.

**Table 2:** Complications and secondary interventions in patients with a simple or complex elbow dislocation that was treated with a hinged fixator

	Total group (*N* = 34)	Simple dislocation (*N* = 9)	Complex dislocation (*N* = 25)
Fixator-related complications			
Pintract infection	6	0	6
Redislocation	1	0	1
Joint incongruency	1	1	0
Muscle hernia	1	0	1
Hardware failure	1	0	1
Total	9* (26%)	1 (11%)	8* (32%)
Fixator-related secondary interventions			
Antibiotics	1	0	1
Incision and drainage	4	0	4
Fixator realignment	2	1	1
Fixator exchange	2	0	2
Total	8* (24%)	1 (11%)	7* (28%)

^*^One patient suffered two complications and two secondary interventions

Non-fixator injury-related complications encountered were elbow stiffness (*n* = 6), ulnar nerve entrapment (*n* = 4), arthrosis (*n* = 2), heterotopic ossification (*n* = 2), joint crepitus for which radial head screws were removed (*n* = 2), wound fistula (*n* = 1), and joint incongruency that was solely related to the trauma and had nothing to do with the hinged external fixator (*n* = 1). For these non-fixator injury-related complications, 11 patients required one or more re-interventions.

An overview of data regarding presence of severe pain and limitations in activities of daily living and work/sports is presented in Table 3. None of the patients reported severe pain at the end of follow-up. Only two patients, with a complex dislocation, required pain medication at discharge. Two patients with a complex dislocation experienced restrictions in ADL and work at the end of follow-up.

**Table 3:** Severe pain and limitations at discharge

	Total group (*N* = 34)	Simple dislocation (*N* = 9)	Complex dislocation (*N* = 25)
Presence of severe pain	0 (0%)	0 (0%)	0 (0%)
Use of pain medication	2 (6%)	0 (0%)	2 (8%)
Limitations in ADL	1 (3%)	0 (0%)	1 (4%)
Limitations in work/sports	1 (3%)	0 (0%)	1 (4%)

The median range of motion of the total cohort at the last patient visit was 140° flexion (P_25_–P_75_ = 120°–145°), 15° constraint in extension (P_25_–P_75_ = 10°–23°), 90° pronation (P_25_–P_75_ = 90°–90°), and 80° supination (P_25_–P_75_ = 56°–90°).

A subanalysis showed that 24 (71%) patients were treated by an experienced surgical team, of which only 3 (13%) developed a fixator-related complication and 3 (13%) underwent a secondary intervention. The other ten (29%) patients of this cohort were operated by a general surgical team: six (60%) of them faced fixator-related complications and five (50%) required a secondary intervention.

### Discussion

The most important finding of this study is that applying a hinged elbow fixator in patients with a simple or complex elbow dislocation yielded—although the injury was severe—excellent range of motion: the median arcs of flexion–extension and pronation-supination were 125° and 170°, respectively. The hinged elbow fixator was applied for both acute management and as revision surgery to treat joint incongruency or a stiff elbow. Retrospectively, 34 patients were included over a period of 12 years, which provides one of the larger cohorts in literature regarding the use of hinged external fixators for elbow dislocations.

Data from this study present excellent outcomes with only a small rate of complications (26%) and few patients with persisting pain or severely limitations (6%), which is in line with current literature. In the series of Hopf et al*.*, one out of 26 patients reported mild resting pain and six patients reported moving pain [12]. Iordens et al. showed median pain scores (VAS) of 0.5 (P_25_–P_75_ = 0.0–1.9) at one year follow-up [3]. Hackl et al*.* showed that early mobilization enables patients to return to work earlier; however, the number of patients limited in their activities of daily living, work or sports were not reported [18].

Due to a lack of comprehensive understanding of the pathogenesis of elbow stiffness, outcomes after the treatment of elbow dislocations are often unsatisfying. The attempt to maintain stability by prolonged immobilization produces a high rate of post-traumatic stiffness [1, 6]. According to several studies, the treatment with a hinged external fixator is an effective supplement to open reduction and internal fixation (ORIF) to improve ligamentous and articular stability as well as the range of motion after an elbow dislocation [6, 7]. Active and passive motion with slow stretching of the elbow during the early postoperative period is crucial in postoperative physical therapy to prevent the emergence of heterotopic ossification, which is seen in up to 75% of the cases [12, 19, 20]. An important determinant for a favorable clinical outcome is the compliance of the patient [2]. If the patient does not cooperate postoperatively, even a good surgical result with anatomic reduction and reconstruction of the joint may lead to an unfavorable clinical result. Therefore, it is important to involve a dedicated rehabilitation team in the postoperative treatment of these patients, who—in our setting—started directly in week 1 after surgery and continued until progression in function was limited.

Because correct mounting the external fixator is technically demanding, the presence of an experienced surgical team was included in the results. In the hands of the experienced surgeon, even complex fracture dislocations of the elbow or their sequelae can be sufficiently reconstructed, which allows a good clinical outcome [1, 14].

A qualitative functional range of motion of the elbow was considered as > 120° flexion and a minimal flexion–extension arc of > 120°. An open arthrolysis, indicated by a stiff elbow, was performed in a patient whose ROM was less than functional. Ahmed & Mistry considered an arc of motion of less than 30° to 130° in a joint that is unresponsive to bracing and therapy as a stiff elbow and therefore as an indication for capsulectomy [19]. Kuhn et al. proposed a capsulolysis if there is a limitation of 30° or more in extension [21]. Treatment with a hinged external fixator was not part of their management. No other studies report on specific ROMs as indication treatment of elbow stiffness.

Other studies regarding hinged external fixators analyzed up to 27 patients [3, 10, 12, 22, 23]. With 34 included patients in the current series, it is among the largest cohort studies to report range of motion for hinged external fixators as a treatment in elbow dislocations to date. Cheung et al*.* included 100 patients, however, they reported only on the complications of hinged external fixation [9].

The hinged external fixator was applied as soon as possible after trauma or after diagnosing persisting instability as a result of extensive arthrolysis for stiff elbow. The fixator was removed after 6–8 weeks, predominantly. This was consistent with other studies [3, 12, 22, 23].

Regarding the range of motion, only one study showed better range in the flexion–extension arc [12]. However, this study exclusively analyzed patients with a simple elbow dislocation. In any of the other published studies, range of motion was inferior to the data presented in this study [3, 10, 20, 23]. The excellent functional results presented, despite serious injury with mostly complex dislocations, might be related to a team with great dedication regarding not only the surgical stabilization of the joint, but also the compliance of postoperative exercises (with the help of a dedicated physical therapist), which is on of the main determinants for a good elbow function. Due to the surgical technique we used, all patients were allowed to have full functional recovery by unrestricted motion exercises postoperatively. The only limitation regarding range of motion was caused by pain, which could be treated sufficiently with painkillers.

The fixator-related complication rate in the current study was 26%, which is low compared with other studies, that report rates ranging between 37 and 57% [3, 10, 22, 23]. Hopf et al. reported a complication rate of 23%, which is comparable to the current results [12]. Only Sakai et al. showed fewer fixator-related complications: 17% [20]. It is tempting to speculate that this might be due to the fact that all procedures were performed by the same surgeon. Cheung et al*.*, who included 100 patients and specifically reported on the complications of hinged external fixation by 2 surgeons, found that 15% of the patients developed minor complications and 10% developed major fixator-related complications [9]. The authors abandoned the use of dynamic external fixators due to these numbers and due to the difficulty of identifying and maintaining the true joint axis.

The possible superior results in the current study together with studies in which only one or two surgeons are involved compared with other studies might suggest that surgeon experience could have contributed to a lower complication rate [9, 20]. This conclusion is supported by the subanalysis that shows less complications in the patients treated by an experienced surgical team. Based on this, results may benefit from centralizing applying a hinged external fixator. A larger hospital volume further reduces morbidity and mortality [24]. Our results hint that an experienced surgical team with better familiarity to this specific treatment can achieve better results.

The most important limitations of the present study are the retrospective nature and the relatively small number of patients included. The follow-up in this study ended when the patient was discharged from outpatient visit. No reliable statements can be done regarding ROM, complications or re-interventions in the period after the last visit to the hospital.

Although we realize that we included a heterogeneous group of patients (acute management of simple and complex elbow dislocations and revision surgery for joint incongruency or stiff elbow), the indication for elbow fixator placement was the same for all patients: creating joint stability and allowing for early active mobilization to improve outcome.

### Conclusion

A hinged elbow fixator applied by a dedicated elbow surgeon in cases of elbow instability after elbow dislocations can result in excellent joint function. Fixator-related complications are mostly mild and only temporary. The results in the current study suggest that surgeon experience may have contributed to a lower complication rate. Based on this, results may benefit from centralizing applying a hinged external fixator.
